# Association between body roundness index and prevalence of kidney stone in the U.S: a study based on the NHANES database

**DOI:** 10.1186/s12894-024-01433-8

**Published:** 2024-04-20

**Authors:** Xike Mao, Yuehan Yang, Junping Yang, Mingwei Chen, Zongyao Hao

**Affiliations:** 1 Department of Urology, the First Affiliated Hospital of Anhui Medical University, Hefei, 230022 Anhui China; 2 Institute of Urology, Anhui Medical University, Hefei, 230022 Anhui China; 3 Anhui Province Key Laboratory of Genitourinary Diseases, Anhui Medical University, Hefei, 230022 Anhui China; 4 Department of General Practice, Wuhu City SecondPeoplès Hospital, Wuhu, Anhui China; 5 Department of Endocrinology, the First Affiliated Hospital of Anhui Medical University, Hefei, Anhui China

**Keywords:** Kidney stones, BRI index, Cross-sectional study, Metabolic syndrome

## Abstract

**Objective:**

This study aimed to evaluate the potential association between the body roundness index (BRI) and kidney stone prevalence in adults in the United States.

**Methods:**

A cohort of participants from the National Health and Nutrition Examination Survey (NHANES) database spanning 2007–2018 were gathered for analysis. Logistic regression analyses, subgroup assessments, and calculations were employed to examine the potential link between BRI and kidney stone prevalence.

**Results:**

The study included 30,990 participants aged > 20 years, of which 2,891 declared a kidney stone history. After modulating all relevant confounding factors, each unit increase in the BRI was linked to a 65% increase in kidney stone prevalence (OR = 1.65, 95% CI: 1.47, 1.85). Sensitivity analyses conducted by categorizing the BRI into three groups revealed a 59% increase in kidney stone prevalence in the highest tertile BRI group compared to the lowest one (OR = 1.59, 95% CI: 1.42, 1.79). Furthermore, dose-response curves depicted a positive near-linear correlation between the BRI and the risk of kidney stone prevalence.

**Conclusion:**

These findings suggest a clinically noteworthy positive correlation between higher BRI values and kidney stone prevalence among the studied US adult population. However, it is essential to acknowledge that the observed relationship does not establish a causal link.

## Introduction

Kidney stones are a prevalent disease in urology, which can result in substantial health consequences, including hydronephrosis, renal impairment, and subsequent renal insufficiency [1]. The global population is experiencing a growing burden of this condition, with a prevalence ranging from 6 to 12%, and its prevalence has shown an upward trend in recent decades, with further increases anticipated in the future [2]. Moreover, there is an increasing recurrence rate of renal stones following the first episode, ranging from 50 to 72%. Epidemiologic surveys have highlighted the frequent and expensive nature of kidney stone treatment in the United States. Nearly 11% of U.S. men and 7% of women disclose a history of at least one kidney stone [3] with a high risk of recurrence. Moreover, the annual cost of treating kidney stones in the U.S. alone exceeds $2 billion [3, 4]. Kidney stones are also a multifactorial disease, influenced by various genetic and environmental factors, including diet, exercise, work environment, and geography [5, 6]. Furthermore, kidney stone formation is associated with several common metabolic disorders, including obesity, diabetes, inflammatory bowel disease, and hypertension [7–10]. Therefore, it is crucial to comprehend the risk factors associated with kidney stone formation as it holds significance in preventing and reducing the cost of treatment.

Obesity poses a significant public health challenge because it is the leading cause of various life-threatening disorders, such as type II diabetes, sleep apnea, hypertension, and heart disease [11–14]. Obesity is a complex and chronic condition influenced by various behavioral, dietary, genetic, socioeconomic, and environmental factors [15]. Epidemiological evidence suggests a potential correlation between the increased prevalence of kidney stones and obesity. The prevalence of urolithiasis in the United States grew from 5.2% in 1988 to 8.8% in 2010, coinciding with an increase in the prevalence of obese patients from 22.5 to 37.4% between 1988 and 2014 [16, 17]. In the majority of studies, body fat is typically evaluated by the measurement of various anthropometric indicators. BMI is the well-documented anthropometric measure applied to determine obesity and overweight in clinical and epidemiological studies [18–20] and is endorsed by the WHO [21]. However, this BMI has limitations in accurately reflecting an individual’s fat distribution or distinguishing between fat mass and muscle weight, which are not relevant factors in assessing kidney stones. In 2013, Thomas DM et al. introduced the BRI [22] as a new predictor of visceral adipose tissue and body fat percentage, which incorporates height and waist circumference (WC) to estimate the percentage of total and localized adiposity, providing a better reflection of the proportion of body and visceral fat better than traditional indices such as BMI, WC, and hip circumference. In this regard, we hypothesized a potential link between BRI and the development of kidney stones. For investigation purposes, we conducted the first cross-sectional study on the association between BRI and kidney stones in a nationally representative survey.

## Materials & methods

### Research population

Data used were obtained from the NHANES database, which is a continuous program administered by the National Center for Health Statistics (NCHS). A complex multistage probability sampling design was selected for the NHANES survey to harvest representative data. All protocols of the NHANES survey are implemented following the U.S. Department of Health and Human Services (HHS) Policy for the Protection of Human Research Subjects and are checked annually by the NCHS Research Ethics Review Board. All subjects involved in the investigation signed informed consent forms. All involved data were freely released by NHANES without additional authorization or ethical review.

**Fig. 1:**
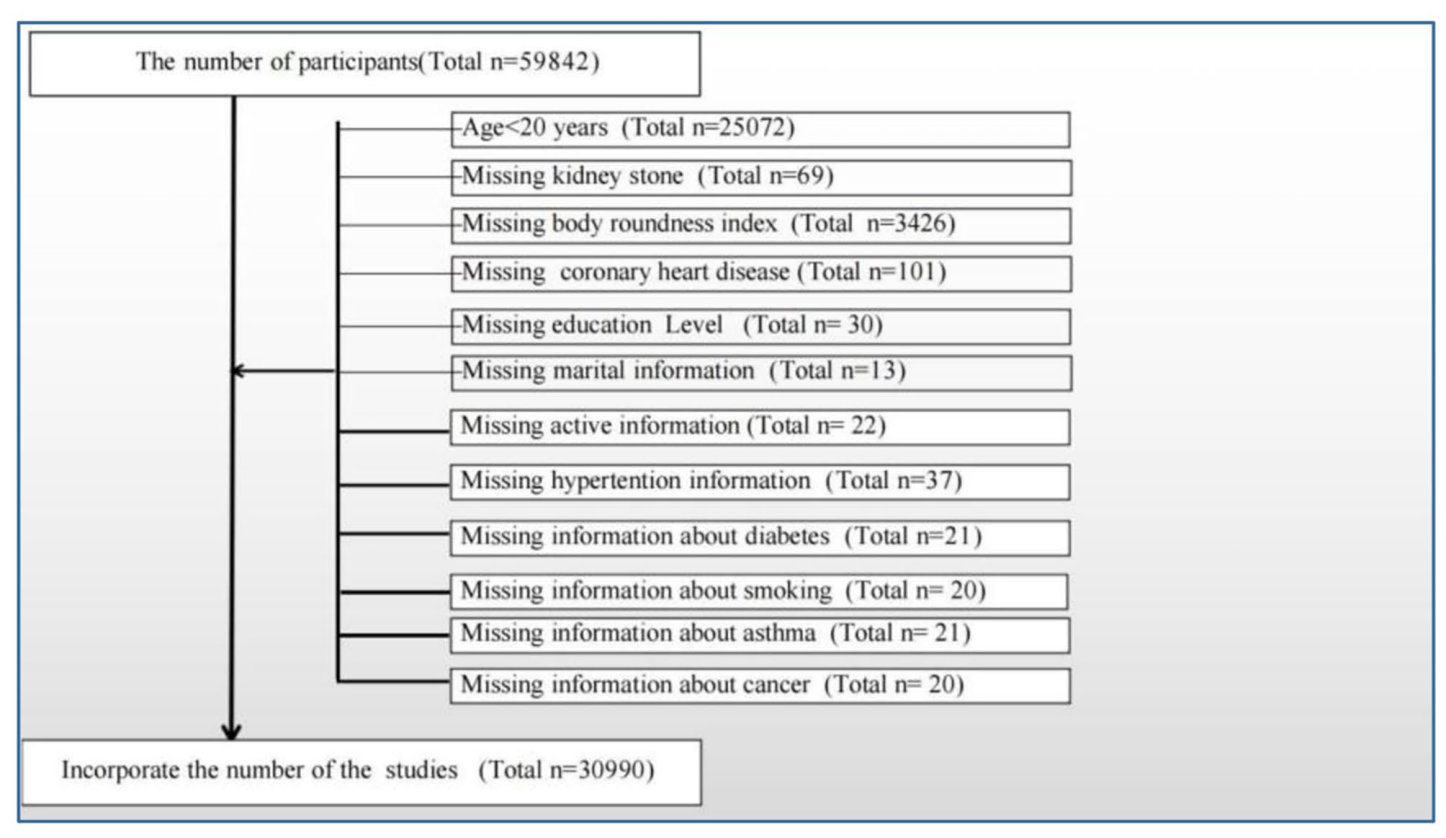
The participants selecting flow chart

For investigation purposes, we pooled publicly available data from participants over 6 survey cycles (2007–2018). A total of 59,842 participants took part in the survey, with only adults being included in this study. Initially, we excluded minors < 20 years of age (*n* = 25,072). After excluding cases with missing data, the final study included a total of 30,990 participants, which included 2,891 participants who self-reported having kidney stones. The specific exclusion criteria are summarized in Fig. 1.

### Data collection

The BRI was developed as an exposure variable in this study, which can be calculated utilizing the formula: BRI = 364.2–365.5 × {1 - [(WC/2π)/(0.5 × height)] 2 } 0.5. To address the right-hand side bias of the BRI data, a natural logarithm (LN) transformation of the BRI was performed to ensure a positively skewed distribution. Then, basic anthropometric data, including weight, body height (BH), and WC, were measured at a mobile examination center with experienced examiners utilizing standardized techniques and equipment. The presence of kidney stones was assessed using questionnaires, with the occurrence of kidney stones serving as an outcome variable. Potential covariates that may confound the linkage between BRI and kidney stones were gathered in multivariate adjusted models. Covariates in our study included sex (male/female), age (years), race, education level, poverty-to-income ratio (PIR), marital status, alcohol consumption, smoking status, physical activity, cholesterol level, uric acid level, hypertension, diabetes, coronary artery disease, cancer, and the METS-IR index, as well as dietary intake factors, including energy, fat, sugar, and water intake. Besides, all participants in the selected years underwent two 24-hour dietary recalls, and the average consumption of the two recalls will be utilized in our analyses. Detailed measurement procedures for the study variables can be assessed publicly at www.cdc.gov/nchs/nhanes/.

### Statistical methods

To guarantee national representativeness, weighted analyses were conducted following the recommended guidelines of the NCHS. The “survey design” R package in R was utilized to address the complex multistage stratified sampling technique employed in NHANES, incorporating the weights provided in the dataset. Continuous variables were expressed as weighted survey means and corresponding 95% confidence intervals (CIs), while categorical variables were expressed as weighted survey and 95% CIs. Survey-weighted linear regression was employed for continuous variables and survey-weighted chi-square tests (for categorical variables) for assessing differences between the two groups. Following the guidelines, multivariate logistic regression models were constructed to explore the BRI, the different BRI tertile groups, and the prevalence of kidney stones in three different models. In Model 1, no adjustment was made for covariates. Model 2 was prepared based on age, sex, race, marital status, and education level. Model 3 was adjusted for all enrolled variables. Additionally, smoothed curve fitting (penalized spline method) and generalized additive model regressions were operated further to assess the relation between BRI and kidney stone prevalence. Inflection point values were yielded following the likelihood ratio test when a nonlinear relationship was identified. Subsequently, multiple regression analyses were implemented, stratified by gender, age, race, hypertension, diabetes, and whether a relative had kidney stones.In the sensitivity analysis, we used ROC curve analysis in order to assess the diagnostic value of BRI in predicting kidney stones in comparison with the traditional BMI. *p* < 0.05 manifested statistically significant. All analyses were completed with the help of the Empower software (www.empowerstats.com; X&Y Solutions, Inc., Boston, MA, USA) and R version 4.0.2 (http://www.R-project.org, The R Foundation).

A missing value treatment was applied by converting continuous variables to categorical variables when they had many missing values, and missing values were self-adjusted to a set of dummy variables.

## Results

The baseline demographic characteristics of the enrolled participants are manifested in Table 1. The LN (BRI) index was 1.73 (1.71, 1.75) in the kidney stone group, which was higher than that of the normal group [1.58 (1.57, 1.59)] (*p* < 0.001).

**Table 1:** Baselines characteristics of participants,weighted

Characteristic	Nonstone formers	Stone formers	*P*-value
	*N* = 28,099	*N* = 2891	
Age(years)	46.49 (46.03 ,46.95)	53.12 (52.52 ,53.73)	< 0.0001
LN(BRI)Index	1.58 (1.57 ,1.59)	1.73 (1.71 ,1.75)	< 0.0001
Gender(%)			< 0.0001
Male	47.74 (47.07 ,48.42)	54.92 (52.33 ,57.49)	
Female	52.26 (51.58 ,52.93)	45.08 (42.51 ,47.67)	
Race(%)			< 0.0001
Mexican American	14.85 (12.99 ,16.93)	11.42 (9.45 ,13.74)	
White	65.07 (62.19 ,67.85)	76.36 (73.23 ,79.22)	
Black	11.77 (10.34 ,13.36)	6.08 (5.08 ,7.27)	
Other Race	8.30 (7.44 ,9.26)	6.14 (4.97 ,7.57)	
Education Level(%)			0.0978
Less than high school	20.53 (19.12 ,22.02)	19.90 (18.01 ,21.94)	
High school	28.80 (27.57 ,30.06)	31.50 (28.84 ,34.28)	
More than high school	50.67 (48.80 ,52.53)	48.60 (45.60 ,51.61)	
Marital Status(%)			< 0.0001
Cohabitation	62.90 (61.64 ,64.14)	69.28 (66.79 ,71.67)	
Solitude	37.10 (35.86 ,38.36)	30.72 (28.33 ,33.21)	
Alcohol(%)			0.8441
Yes	60.60 (59.13 ,62.06)	59.95 (56.95 ,62.88)	
No	18.57 (17.51 ,19.68)	19.23 (17.08 ,21.58)	
Unclear	20.83 (19.72 ,21.97)	20.82 (18.22 ,23.68)	
High Blood Pressure(%)			< 0.0001
Yes	29.78 (28.79 ,30.79)	46.23 (43.36 ,49.14)	
No	70.22 (69.21 ,71.21)	53.77 (50.86 ,56.64)	
Diabetes(%)			< 0.0001
Yes	8.57 (8.09 ,9.07)	17.57 (15.92 ,19.35)	
No	91.43 (90.93 ,91.91)	82.43 (80.65 ,84.08)	
Smoked(%)			0.0001
Yes	43.58 (42.35 ,44.81)	49.28 (46.46 ,52.10)	
No	56.42 (55.19 ,57.65)	50.72 (47.90 ,53.54)	
Physical Activity(%)			0.0015
Never	26.39 (25.42 ,27.39)	30.48 (28.33 ,32.72)	
Moderate	31.90 (30.94 ,32.87)	31.09 (28.82 ,33.45)	
Vigorous	41.71 (40.59 ,42.84)	38.43 (35.85 ,41.08)	
Asthma(%)			0.0022
Yes	85.47 (84.78 ,86.14)	82.66 (80.75 ,84.43)	
No	14.53 (13.86 ,15.22)	17.34 (15.57 ,19.25)	
Coronary Artery Disease(%)			< 0.0001
Yes	3.02 (2.66 ,3.43)	6.33 (5.30 ,7.55)	
No	96.98 (96.57 ,97.34)	93.67 (92.45 ,94.70)	
Cancers(%)			< 0.0001
Yes	9.49 (8.99 ,10.01)	15.70 (14.17 ,17.36)	
No	90.51 (89.99 ,91.01)	84.30 (82.64 ,85.83)	
PIR(%)			0.1121
<1.3	20.30 (19.05 ,21.62)	18.32 (16.59 ,20.18)	
≥ 1.3<3.5	32.52 (31.29 ,33.77)	34.93 (32.43 ,37.52)	
≥ 3.5	39.61 (37.77 ,41.47)	39.75 (36.56 ,43.02)	
Unclear	7.57 (6.93 ,8.27)	7.00 (5.77 ,8.47)	
Serum Cholesterol(%)			0.0332
Lower	46.40 (45.41 ,47.40)	48.53 (46.08 ,50.98)	
Higher	49.12 (48.07 ,50.16)	48.11 (45.81 ,50.43)	
Unclear	4.48 (4.07 ,4.93)	3.36 (2.60 ,4.33)	
Serum Uric Acid(%)			< 0.0001
Lower	46.58 (45.75 ,47.41)	40.43 (38.05 ,42.86)	
Higher	48.93 (47.96 ,49.91)	56.20 (53.59 ,58.77)	
Unclear	4.49 (4.07 ,4.94)	3.37 (2.61 ,4.34)	
METS-IR(%)			< 0.0001
Lower	41.91 (40.68 ,43.15)	30.65 (28.30 ,33.10)	
Higher	37.41 (36.07 ,38.77)	46.34 (43.53 ,49.17)	
Unclear	20.68 (19.07 ,22.38)	23.01 (20.28 ,25.98)	
Serum Creatinine(%)			< 0.0001
Lower	46.89 (45.90 ,47.87)	39.73 (37.05 ,42.48)	
Higher	48.64 (47.59 ,49.70)	56.96 (54.26 ,59.61)	
Unclear	4.47 (4.06 ,4.92)	3.31 (2.56 ,4.27)	

For continuous variables: survey-weighted mean (95% CI), *P*-value was by survey-weighted linear regression (svyglm)

For categorical variables: survey-weighted percentage (95% CI), *P*-value was by survey-weighted Chi-square test (svytable)

### Higher BRI was associated with a higher prevalence of kidney stones

The analysis revealed a positive correlation between LN (BRI) index and kidney stone prevalence. The fully adjusted model (model 3) consistently showed this positive association (OR = 1.65, 95% CI: 1.47, 1.85), suggesting that each unit increase in LN (BRI) index shared linkage with a 65% increased risk of kidney stones. Additionally, sensitivity analysis (Table 2) revealed a significant 59% increase in the likelihood of kidney stone occurrence was observed in Tertile 3 compared with the lowest tertile of the lowest LN (BRI) index (Tertile 1) (OR = 1.59, 95% CI: 1.42, 1.79), respectively.

**Table 2:** Logistic regression analysis between BRI index with kidney stone prevalence

Characteristic	Model 1 OR(95%CI)	Model 2 OR(95%CI)	Model 3 OR(95%CI)
LN(BRI)Index	2.18 (1.99, 2.40)	2.01 (1.81, 2.23)	1.65 (1.47, 1.85)
Categories			
Tertile 1	1	1	1
Tertile 2	1.75 (1.58, 1.95)	1.46 (1.31, 1.63)	1.37 (1.23, 1.53)
Tertile 3	2.22 (2.00, 2.45)	1.91 (1.72, 2.13)	1.59 (1.42, 1.79)
P for trend	< 0.01	< 0.01	< 0.01

Model 1 was adjusted for no covariates;

Model 2 was adjusted for age,gender, race,marital status and education;

Model3 was adjusted for covariates in Model 2 + diabetes,blood pressure,PIR,total water,total kcal,total sugar,total fat,smoked,physical activity,alcohol use,serum cholesterol,serum uric acid,coronary artery disease, serum creatinine,METS-IR index and cancers were adjusted

### Dose-response and threshold effect analyses of BRI on kidney stone prevalence

**Fig. 2:**
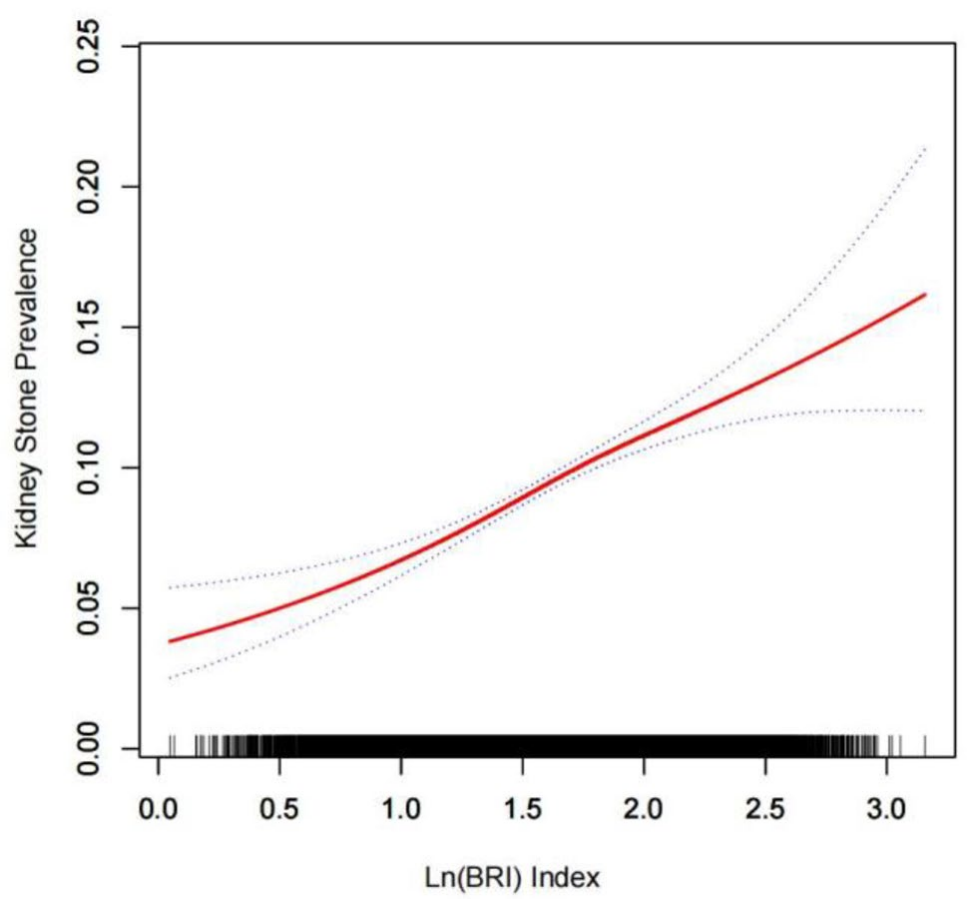
Density dose-response relationship between BRI index with kidney stone prevalence. The area between the upper and lower dashed lines is represented as 95% CI. Each point shows the magnitude of the BRI index and is connected to form a continuous line. Adjusted for all covariates except effect modifier

The relation between BRI and kidney stones was further substantiated by employing generalized additive modeling and smoothed curve fitting. Our results revealed an approximately linear positive correlation between LN (BRI) index and kidney stones (Fig. 2).

### Subgroup analysis

Subgroup analysis was implemented to test the robustness of the association between LN (BRI) and kidney stones. Results were shown in the following: Male group (OR = 1.63, 95% CI: 1.37, 1.93), female group (OR = 1.52, 95% CI: 1.29,1.79), age < 40 years group (OR = 1.29, 95% CI: 1.03, 1.60), age 40–59 years group (OR = 1.79, 95% CI: 1.47, 2.19), age ≥ 60 group (OR = 1.60, 95% CI:1.32, 1.94), Mexican American group (OR = 1.99, 95% CI: 1.08, 1.80), White group (OR = 1.65, 95% CI: 1.41, 1.94), Black group (OR = 1.62, 95% CI: 1.23, 2.14), Others group (OR = 2.35, 95% CI: 1.57, 3.52), hypertensive group (OR = 1.43, 95% CI: 1.020, 1.171), non-hypertensive group (OR = 1.73, 95% CI: 1.48, 2.02), diabetic group (OR = 1.60, 95% CI: 1.20, 2.13), and non-diabetic group (OR = 1.64, 95%CI: 1.44, 1.86) (Table 3).

**Table 3:** Subgroup analysis between BRI index with kidney stone prevalence

Characteristic	Model 1 OR(95%CI)	Model 2 OR(95%CI)	Model 3 OR(95%CI)
Stratified by gender			
Male	2.81 (2.45, 3.22)	1.98 (1.70, 2.31)	1.63 (1.37, 1.93)
Female	2.00 (1.74, 2.29)	1.95 (1.69, 2.25)	1.52 (1.29, 1.79)
Stratified by race			
Mexican American	1.90 (1.53, 2.36)	1.65 (1.30, 2.09)	1.39 (1.08, 1.80)
White	2.24 (1.96, 2.55)	2.02 (1.76, 2.33)	1.65 (1.41, 1.94)
Black	1.95 (1.56, 2.45)	1.83 (1.42, 2.35)	1.62 (1.23, 2.14)
Other Race	3.53 (2.52, 4.94)	3.22 (2.24, 4.63)	2.35 (1.57, 3.52)
Stratified by age(years)			
20–39	1.73 (1.44, 2.07)	1.69 (1.39, 2.04)	1.29 (1.03, 1.60)
40–59	2.13 (1.81, 2.52)	2.35 (1.97, 2.80)	1.79 (1.47, 2.19)
60–85	1.73 (1.46, 2.04)	1.93 (1.62, 2.30)	1.60 (1.32, 1.94)
Stratified by hypertension			
Yes	1.49 (1.28, 1.74)	1.62 (1.38, 1.91)	1.43 (1.20, 1.71)
No	2.13 (1.87, 2.43)	1.93 (1.67, 2.23)	1.73 (1.48, 2.02)
Stratified by diabetes			
Yes	1.54 (1.20, 1.97)	1.77 (1.35, 2.31)	1.60 (1.20, 2.13)
No	2.01 (1.80, 2.23)	1.84 (1.63, 2.06)	1.64 (1.44, 1.86)

Model 1 = no covariates were adjusted

Model 2 = Model 1 + age,gender, race,marital status and education were adjusted

Mode3 = adjusted for all covariates except effect modifier

### Sensitivity analysis

**Fig. 3:**
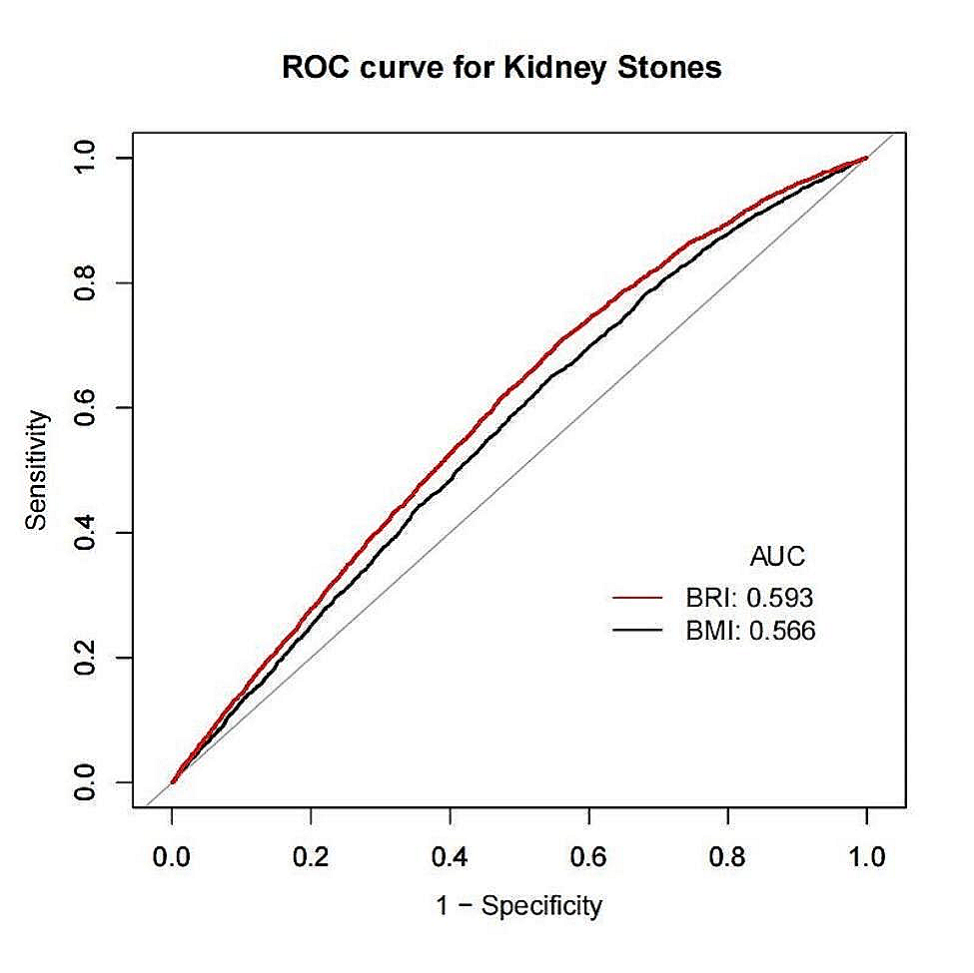
Diagnostic performance of obesity BMI and BMI index on kidney stone prevalence

Next, we plotted ROC curves to compare the diagnostic effect of BMI and BRI index on the prevalence of kidney stones. The analysis showed that the diagnostic effect of both BMI and BRI on the prevalence of kidney stones was statistically significant (AUC > 0.5) (Fig. 3). In addition, the area under the ROC curve was higher for BRI than for BMI (AUC = 0.59).

## Discussion

This study is a comprehensive nationwide investigation examining the impact of various obesity indices on urate levels, hyperuricemia, and gout. Our findings reveal that BMI, BRI, and WWI exhibited positive associations with elevated urate levels, hyperuricemia, and gout incidence, respectively. Notably, BRI and WWI, which specifically measure central obesity, demonstrated higher sensitivity in predicting these conditions compared to BMI alone.These results highly documented the potential of BRI as a predictor of kidney stone development.

Obesity and kidney stones are significant public health concerns worldwide. Previous studies have noted a significant association between obesity and an increased prevalence and recurrence of kidney stones [23–25]. Most previous studies have primarily concentrated on the relation between BMI, the traditional obesity index, and kidney stones. A recent study has discovered that short height links with stone formation independent of body weight [26]. This phenomenon can be explained by the limitation of BMI [27] and the existence of the obesity paradox [28]. Some recent studies investigating the correlation between obesity and kidney stones have favored the use of non-traditional obesity indicators as a measure of obesity, such as the Visceral adiposity Index [29], Android to Gynoid ratio [30], and BRI.

BRI, as a newly developed obesity index, estimates the percentage of total and localized fat based on height and WC, which weakens the influence of BMI to a certain extent and provides a more accurate reflection of body fat better than the traditional indices such as BMI, WC, and hip circumference. BRI specifically focuses on real central obesity, which is not solely dependent on body weight. Researchers have shown increasing interest in the correlation between increased visceral fat associated with central obesity and adverse metabolic features [31]. Currently, BRI has been extensively explored in many fields, including coronary heart disease, carotid atherosclerosis, and diabetes mellitus [32–34]. Our results confirmed a significant correlation between the BRI and the prevalence of kidney stones. To identify specific populations for the BRI and improve kidney stone prevention, a subgroup analysis was performed. In our gender analysis, the effect of BRI on the prevalence of kidney stones was lower in the female group than in the male group. Many studies have found a gradual reduction in the gender disparity regarding the risk of kidney stones [35, 36], and our results are basically in line with these previous reports. In the age subgroup analysis, we found a weaker correlation between a high BRI and the prevalence of kidney stones among respondents aged under 39 compared to those aged 39 and above. This encouraging finding highlights the significance of managing and controlling BRI in middle and old ages, as it can better prevent the occurrence of kidney stones in middle-aged and older adults. This result is similar to that reported by Shavit et al. [37]. Among the gender subgroups, the black and white groups exhibited the weakest association between the BRI and the prevalence of kidney stones, potentially due to their lower susceptibility to obesity-related effects than other racial groups [38, 39]. In the hypertension and diabetes stratification, we found an interesting phenomenon that there was a stronger association between BRI and kidney stone prevalence in non-hypertensive and non-diabetic populations, which was similarly reported by Zheng’s [40] and shen et al.‘s [41], supporting the validity of our results. However, further studies are still needed to confirm the causality in prospective cohort studies.

The potential mechanisms concerning the correlation between obesity and kidney stones have not been fully elucidated. The following are a few plausible relationships that have been reported so far that may exist between them. One viewpoint suggests that obese patients often experience excessive fat deposition in the liver, disrupting purine metabolism and resulting in increased production and excretion of uric acid, ultimately leading to a higher prevalence of uric acid stones [42, 43]. Secondly, obesity can induce insulin resistance, impair ammonia excretion, and subsequently elevate uric acid levels. Moreover, insulin resistance can also facilitate the uptake of citrate in the renal tubules, leading to a decrease in urinary citrate content, which is also a critical risk factor for calcium stone formation [41]. Thirdly, obese patients experience alterations in lipid metabolism, which can affect the biological function of renal tubular epithelial cells in many ways. These abnormalities in lipid metabolism have been implicated in diverse renal disorders and contribute to the development of renal stone disease [44, 45]. In addition, adipocytes themselves produce various adipokines, most notably hypolipocalcemia [46], due to their secretory properties. Besides, they also increase the production of reactive oxygen species [47, 48], which can damage renal tubular epithelial cells. Furthermore, renal cell damage and inflammation may cause idiopathic stone disease [49].

Our study still has some shortcomings: (1) It was based on a cross-sectional design, limiting our ability to determine the causal relationship between BRI and the prevalence of kidney stones. (2) Although adjusting for possible covariates, confounding from unknown variables remains a possibility. (3) Kidney stone variables were obtained from questionnaires, introducing recall bias, and some asymptomatic kidney stones may also have influenced our results.

## Conclusion

This study highlights a potential association between elevated BRI levels and an increased risk of kidney stones. It also suggests that obesity management, as assessed by BRI, may be beneficial to kidney health, especially in middle-aged and older adults. Nonetheless, further studies are still needed to substantiate our findings.

## Data Availability

We will provide raw data upon request.

## Declarations

### Statement of agree publication

All authors have read this manuscript and agree to publish this manuscript.
